# Mutual inhibition effect of sulfur compounds in the hydrodesulfurization of thiophene, 2-ethylthiophene and benzothiophene ternary mixture

**DOI:** 10.1038/s41598-021-98552-1

**Published:** 2021-09-24

**Authors:** Catalina Calin, Cristian Leostean, Ancuța Roxana Trifoi, Elena-Emilia Oprescu, Elizabeth Wiita, Ionut Banu, Rami Doukeh

**Affiliations:** 1 Petroleum-Gas University of Ploiești, Bucharest Blvd. 39, 100680 Ploiești, Romania; 2 National Institute for Research and Development of Isotopic and Molecular Technologies, 67-103 Donat, 400293 Cluj-Napoca, Romania; 3 Faculty of Material Science and Engineering, University Politehnica of Bucharest, 313 Splaiul Independentei, 060042 Bucharest, Romania; 4 National Institute for Research and Development for Chemistry and Petrochemistry ICECHIM Bucuresti, 202 Splaiul Independentei, 060021 Bucharest, Romania; 5 Columbia University, 3009 Broadway, New York, NY 10027 USA; 6 Department of Chemical and Biochemical Engineering, University Politehnica of Bucharest, 1-7 Polizu Str., 011061 Bucharest, Romania

**Keywords:** Diesel fuel, Chemical engineering

## Abstract

The hydrodesulphurization of a model component and equimolar ternary mixture of thiophene, 2-ethylthiophene and benzothiophene over sulphided CoMo/γ-Al_2_O_3_ and CoMo/γ-Al_2_O_3_-Nb_2_O_5_ catalysts were investigated in a fixed bed flow reactor. The catalysts were prepared by incipient wetness impregnation method and characterized by textural characteristics, total acidity and chemical species present on the catalysts surface. The characterization results showed that both CoMo/γ-Al_2_O_3_-Nb_2_O_5_ and sulphided CoMo/γ-Al_2_O_3_ catalysts exhibit a mesoporous structure with cylindrical pores open at both ends, evidenced by the IV type adsorption–desorption isotherm with a H1 hysteresis loop and have an average pore diameter between 3 and 4 nm. The chemical species present on the catalysts surface evaluated by XPS indicated that Co^2+^ and Mo^4+^ species are present in the sulfide form on both catalysts surfaces. In addition, there are also found oxidic species arising from incomplete reduction and sulphidation. The presence of niobium oxide in the catalytic support had a positive effect in leading to higher specific surface area (170 m^2^/g) and total acidity (0.421 meq/g) compared with CoMo/γ-Al_2_O_3_ catalyst (140 m^2^/g and 0.283 meq/g) respectively. The evaluation results from the hydrodesulfurization showed that CoMo/γ-Al_2_O_3_-Nb_2_O_5_ catalyst had a higher activity in hydrodesulphurization process of thiophene, 2-ethylthiophene and benzothiophene. The CoMo/γ-Al_2_O_3_-Nb_2_O_5_ catalyst eliminated sulfur from the single component feed (corresponding to 2380 ppm S) and reduced below 10 ppm, for the feed consisting in the equimolar ternary mixture of thiophene, 2-ethylthiophene and benzothiophene (2380 ppm S). The reactivity of thiophen compounds was reduced due to competitive adsorption. It was observed that benzothiophene inhibits the transformation of thiophene and 2-ethylthiophene. A mutual inhibition effect between sulfur compounds was also observed when thiophene, 2-ethylthiophene and benzothiophene were combined and tested over the CoMo/γ-Al_2_O_3_ catalyst. The inhibition effect had a lower intensity by introducing Nb_2_O_5_ in the catalyst support.

## Introduction

The sulfur content of petroleum ranges from 0.1 to 8%^1^, but normally does not exceed 1%. The distribution of sulfur compounds in petroleum distillates, such as, mercaptans, sulfides, thiophene, benzothiophene, dibenzothiophene, etc. depends on the boiling temperature. The most important aromatic sulfur compounds in gasoline are thiophene, alkylthiophene, and benzothiophene. These are commonly found in kerosene and gas oil^2^. Removal of sulfur from petroleum fractions is of particular interest because sulfur compounds poison the catalysts used in the processing of these fractions, such as catalytic cracking and catalytic reforming. Environmentally, it is critical to minimize sulfur dioxide emissions, from the combustion of sulfur compounds in fuels, as they have many harmful properties. Furthermore, many countries have imposed strict legislative limits regarding sulfur content in fuels, aiming to produce cleaner fuels with less than 10 ppm sulfur contents. In the catalytic hydrodesulfurization (HDS), the organosulfur compounds are usually converted to hydrocarbons and hydrogen sulfide which is then catalytically oxidized by air to produce elemental sulfur^3^. HDS usually takes place at elevated temperatures, up to 370 °C and pressures from 20 to 60 bar^4^, depending on the type of petroleum fraction (gasoline, kerosene, gas oil) and the desired degree of organic sulfur removal.

The most important industrial catalysts for HDS are commonly composed of Mo(W) and Co(Ni) sulfides supported on a highly porous γ-Al_2_O_3_^5^. Catalytic supports such as mesoporous materials, zeolites^6, 7^, carbon^8^, clays^9^ have also been studied. The support is very important for the catalyst properties, as it can affect its aging, deactivation, regeneration, reducibility, ease of sulphidation and catalytic recovery^10^. Benzothiophene HDS on Co-Mo catalysts, with varying Co/Mo molar ratios in a batch system has been studied at 275 °C and 40 bar. After 5 h, the conversion of benzothiophene on Mo-S was 76.9% and increased to 93.9% with a 0.3 molar ratio of Co-Mo-S but decreased to 61.1% at a 0.5 molar ratio of Co-Mo-S^2^. The catalytic activities of zeolite supported Mo and Mo-Ni catalysts for benzothiophene HDS, were measured at 300 °C and 30 bar in a flow system. For NiMo catalyst, the highest conversion of benzothiophene was 94%^11^. Niobium pentoxide has also been reported as an efficient catalyst support, alone and in combination with γ-alumina, for Mo and Ni-based HDS catalysts^12–15^. Niobia-alumina mixed oxide supports have been studied for diesel HDS at 300 °C and 55 bar, using a NiMo catalyst. Nb_2_O_5_, single oxide supported catalysts had greater sulfur removal, but the highest activities of both sulfur and nitrogen removal occurred with a 78 (wt%) Nb_2_O_5_. Sulfur removal rate of this catalyst was about 1.4 times greater that of the Al_2_O_3_, supported catalyst^13^. Contrary to this study, Rocha et al. affirmed that the addition of 20% Nb_2_O_5_ to γ-Al_2_O_3_ deactivates the NiMo catalyst^14^, while for the Mo/γ-Al_2_O_3_ catalyst, the presence of 20% Nb_2_O_5_ has a positive influence on thiophene HDS^15^. Furthermore, Faro et al.^16^, reported that in HDS of thiophene, activity of the niobia-supported nickel catalysts was much higher than the activity of the alumina-supported ones, but the activity of the niobia-supported molybdenum catalysts was lower than that of the alumina-supported catalyst. Despite the large number of published studies regarding the individual HDS of organic sulfur compounds, petroleum fractions contain different sulfur compounds that can influence each other in the HDS process. For example, primary gasoline from catalytic cracking has to be hydro-sulfurized because it still contains sulfur compounds, including thiophene and its alkylated derivatives, as well as benzothiophene^17^. In this context, it is surprising that the mutual influence of sulfur compounds in the HDS process has not been intensively studied, despite the importance of this process. The inhibition effect of a mixture of organic sulfur compounds in HDS, was studied by Brunet et al.^18^. In the mentioned study, the mutual inhibition effect was reported for the HDS of the binary mixture of benzothiophene and 2-methylthiophene, at 250 °C and 20 bar.

This paper aims to compare the activity of Co-Mo catalysts supported both on classical alumina support and alumina modified with niobium oxide. Furthermore, this research studies the inhibition effect in HDS for a mixture of aromatic sulfur compounds, depending on their structure and reaction conditions. For this purpose, thiophene (T), 2-ethylthiophene (2-ET) and benzothiophene (BT) were selected because they have different aromatic character due to the density of π electrons in their aromatic rings. To study the mutual inhibition effect changes, when a small amount of Nb_2_O_5_ is added to γ-Al_2_O_3_ support two catalysts, CoMo/γ-Al_2_O_3_ and CoMo/γ-Al_2_O_3_-Nb_2_O_5_ were tested over HDS of individual thiophene, 2-ethylthiophene and benzothiophene and their equimolar ternary mixture.

## Experimental

### Materials

Thiophene (99%), 2-ethylthiophene (97%), benzothiophene (98%), ammonium heptamolybdate (NH_4_)_6_Mo_7_O_24_, 99%), cobalt (II) nitrate hexahydrate (Co(NO_3_)_2_·6H_2_O, 98%), Nb_2_O_5_ (99.99%), HNO_3_ (70%), dimetyhldisulphide (> 98%), heptane (99%) and diethylamine (> 99.5) were purchased from Sigma Aldrich; γ-Al_2_O_3_ (V-250) was purchased from Euro Support, H_2_ (99.9%), N_2_ (100%) and He (99.999%) were purchased from Linde Gas. All reagents were used without further purification.

### Catalysts preparation and characterization

The γ-Al_2_O_3_ and γ-Al_2_O_3_-Nb_2_O_5_ supports were prepared by mixing the commercial powder γ-alumina or γ-alumina and Nb_2_O_5_ with 10 wt% nitric acid, for 3 h at ambient temperature. The resulting mixture was extruded into cylinders with the average size of 1 mm and dried in air for 48.0 h and further dried at 160 °C for 6.0 h. Calcination step was carried out at 450 °C (10 °C/min) for 8.0 h. The niobium concentration in γ-Al_2_O_3_-Nb_2_O_5_ support was 5%. The catalysts used in the present study were prepared by sequential impregnation. Aqueous solutions of ammonium heptamolybdate ((NH_4_)_6_Mo_7_O_24_) and cobalt (II) nitrate hexahydrate (Co(NO_3_)_2_·6H_2_O) were used. The heptamolybdate ((NH_4_)_6_Mo_7_O_24_) was first dissolved in 10.0 wt% ammonia water. The solution was loaded on the supports (γ-Al_2_O_3_ or γ-Al_2_O_3_-Nb_2_O_5_) using incipient wetness impregnation method. The solid was first dried at room temperature for 24.0 h and then at 120 °C for 4.0 h. The calcination was done at 450 °C, for 2.0 h. The cobalt (II) nitrate hexahydrate (Co(NO_3_)_2_·6H_2_O) was dissolved in deionized water, and then the solution was loaded on the Mo/Al_2_O_3_ (Mo/γ-Al_2_O_3_-Nb_2_O_5_). The materials were dried in air for 48.0 h, dried at 120 °C for 4.0 h, and calcined at 450 °C for 2.0 h, thus resulting in CoMo oxidized HDS catalysts. The mass fractions of Mo and Co were 8.0% and 4.0%, respectively, for each catalyst, and the Nb_2_O_5_ mass fraction represented 5.0% of the support. Before the experimental tests of HDS, the catalysts were reduced to metal form with hydrogen, for 2.0 h (250 °C, 5 bar); then the temperature was increased to 450 °C, and the reduction continued for 6.0 h. After reduction, the catalysts were activated (pre-sulfided) for 8.0 h (250 °C, 5 bar) in a solution of 1% dimethyl-disulphide solution in heptane (flowrate 1 mL/min). Both catalysts have been characterized before and after sulphidation. Textural characteristics of the catalysts (surface area, pore volume, mean pore diameter, pore-size-distribution) were determined by nitrogen adsorption at low temperature, using an Autosorb 1 Quantacrome instrument. The samples were vacuum-dried before the adsorption at 250 °C for 3 h.

The total acidity of the solid catalysts was measured by temperature-programmed desorption of diethylamine (DEA-TPD) method^19^, carried out by heating the samples from 20 to 600 °C, in nitrogen atmosphere with 10 °C/min, using a DuPont Instrument Thermal Analyst 2000/2100 coupled to a module 951 Thermogravimetric Analyzer. The total acidity was determined based on the diethylamine desorbed on the acidic sites, expressed as miliequivalents DEA per gram of catalyst. The pyridine-adsorbed FT-IR spectrum was recorded using Jasco 610 spectrometer, with a scanning range from 4000 to 400 cm^−1^, a scan rate of 4 cm^−1^·s^−1^ and an average of 64 measurements in the final spectrum.

The X-Ray Photoelectron Spectroscopy (XPS) spectra were obtained on a SPECS spectrometer by using the Al anode (1486.6 eV) radiation.

### Catalytic activity

The catalytic HDS tests of thiophene (T 0.625 wt%) 2-ethylthiophene (2-ET 0.835 wt% ) or benzothiophene (BT 1 wt%) or their equimolar mixture (T 0.21 wt%, 2-ET 0.28 wt% and BT 0.33 wt%) diluted in n-hexane over sulfided CoMo/γ-Al_2_O_3_ and CoMo/γ-Al_2_O_3_-Nb_2_O_5_ catalysts, have been performed in a fixed bed flow reactor described in our previous work^19^. The experiments were carried out at a pressure of 30 bar, a liquid hourly space velocity (LHSV) of 2 h^−1^, an H_2_/sulfur compounds molar ratio of 60/1 and temperatures in the range 200–275 °C. The raw materials and the reaction mixtures were analyzed by Gas Chromatography (Varian 3800) coupled with Mass Spectrometry (Varian 4000), equipped with Agilent VF-5ms capillary column (L = 30 m, D = 250 μm, d = 0.25 μm). The sulfur content for the feed (reactants) and reactor effluent was calculated based on the concentrations of the sulfur compounds from GC–MS analyses. All experiments were performed in triplicate.

## Results and discussion

### Catalysts characterization

#### Textural characteristics of the catalysts

**Figure 1:**
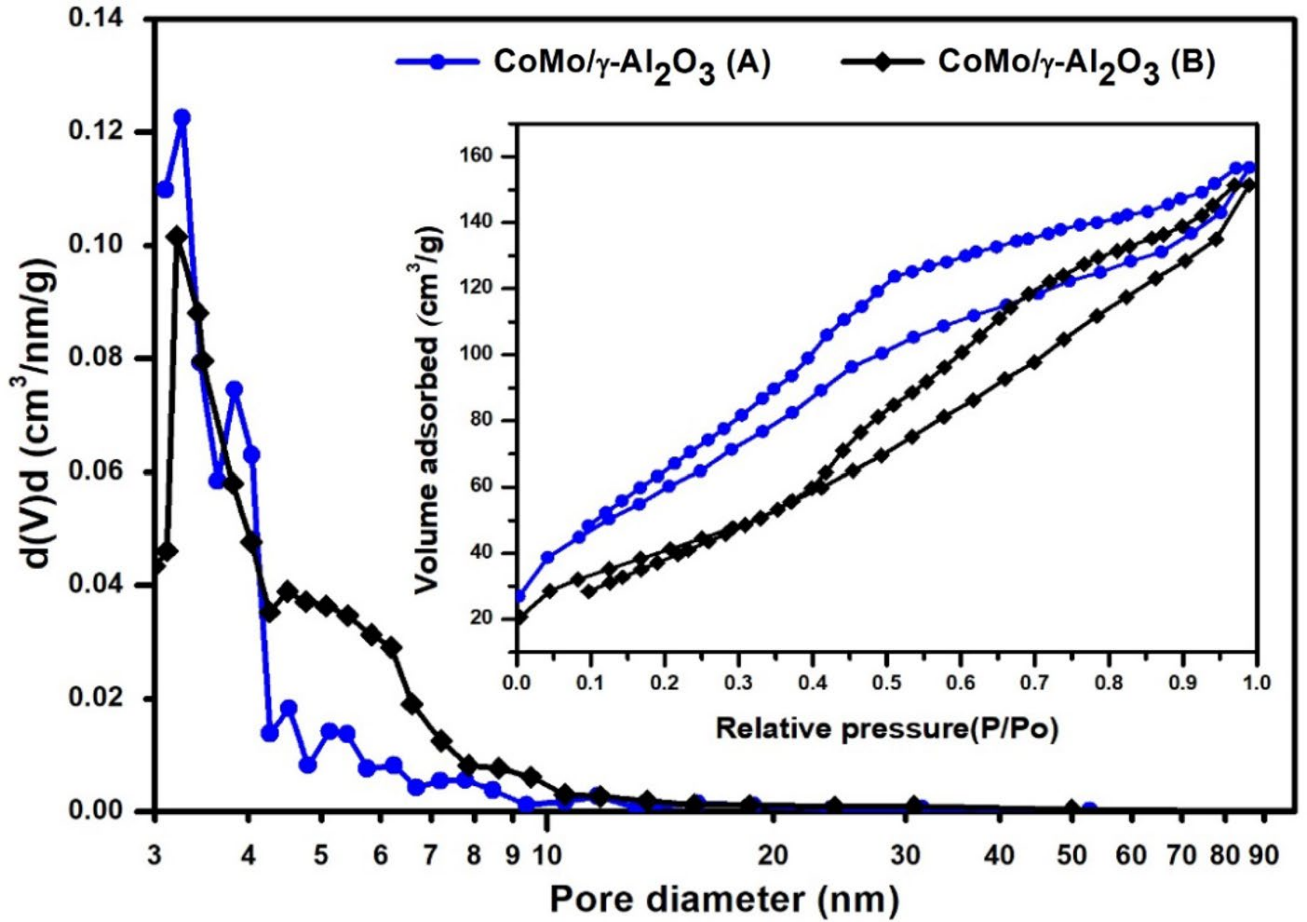
Pore size distribution and adsorption desorption isotherm of the CoMo/γ-Al_2_O_3_ catalyst before (**A**) and after (**B**) sulphidation.

**Figure 2:**
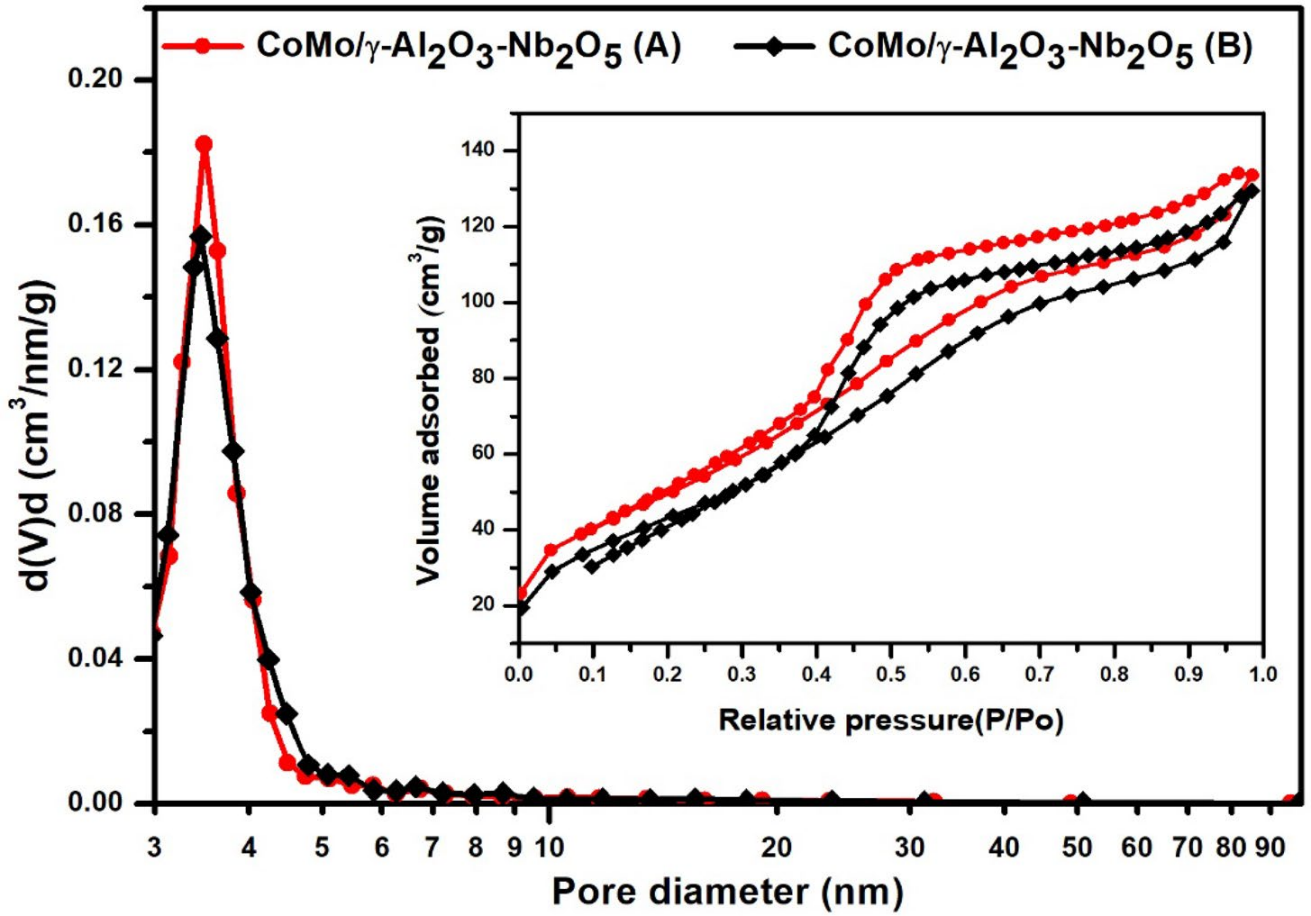
Pore size distribution and adsorption desorption isotherm of the CoMo/γ-Al_2_O_3_-Nb_2_O_5_ catalyst before (**A**) and after (**B**) sulphidation.

The pore size distribution, calculated from the BJH desorption isotherms and the adsorption/desorption isotherm of the CoMo/γ-Al_2_O_3_ and CoMo/γ-Al_2_O_3_-Nb_2_O_5_ catalysts are presented in Figs. 1 and 2. The textural properties of the catalysts and the supports are listed in Table 1. As can be seen from Figs. 1 and 2, both catalysts, CoMo/γ-Al_2_O_3_ and CoMo/γ-Al_2_O_3_-Nb_2_O_5_, exhibit a mesoporous structure with cylindrical pores open at both ends, evidenced by the IV type adsorption–desorption isotherm with a H1 hysteresis loop and have an average pore diameter between 3–4 nm. The presence of niobium pentoxide into the γ-Al_2_O_3_ support decreased the surface area from 230 to 192 m^2^/g and pore volume from 0.496 to 0.199 cc/g. The mean pore diameter of both supports is also between 3 and 4 nm. Before sulphidation, CoMo/γ-Al_2_O_3_-Nb_2_O_5_ had a specific area of 176 m^2^/g, compared to that of CoMo/γ-Al_2_O_3_ which had a specific area of 184 m^2^/g. However, after sulphidation, the specific area of the CoMo/γ-Al_2_O_3_-Nb_2_O_5_ catalyst decreased to 170 m^2^/g, while the specific area of CoMo/γ-Al_2_O_3_ decreased to 140 m^2^/g. After sulphidation the specific area of CoMo/γ-Al_2_O_3_-Nb_2_O_5_ catalyst was higher than that of CoMo/γ-Al_2_O_3_. It is also noted that the pore volume decreased greatly in the case of the CoMo/γ-Al_2_O_3_ catalyst, from 0.238 cc/g before sulphidation to 0.164 cc/g after sulphidation, while for the CoMo/γ-Al_2_O_3_-Nb_2_O_5_ catalyst, the pore volume decreased less, from 0.192 to 0.180 cc/g. A significant decrease of surface area for sulfide CoMo/γ-Al_2_O_3_ catalyst may be due to sintering of the metal species. In the case of CoMo/γ-Al_2_O_3_-Nb_2_O_5_ catalyst, the metal-support interaction is stronger and the sintering process has a lower influence.

**Table 1:** Textural properties of the support and catalysts.

Catalyst	Surface area (m^2^/g)		Pore volume (cc/g)		Mean pore diameter Dv(d) (nm)	
	A*	B*	A*	B*	A*	B*
γ-Al_2_O_3_	230	–	0.496		3.74	–
γ-Al_2_O_3_-Nb_2_O_5_	192	–	0.199		3.65	–
CoMo/γ-Al_2_O_3_	184	140	0.238	0.164	3.44	3.27
CoMo/γ-Al_2_O_3_-Nb_2_O_5_	176	170	0.192	0.180	3.46	3.37

*A—samples before sulphidation.

*B—samples after sulphidation.

#### Acidity of the catalysts

**Figure 3:**
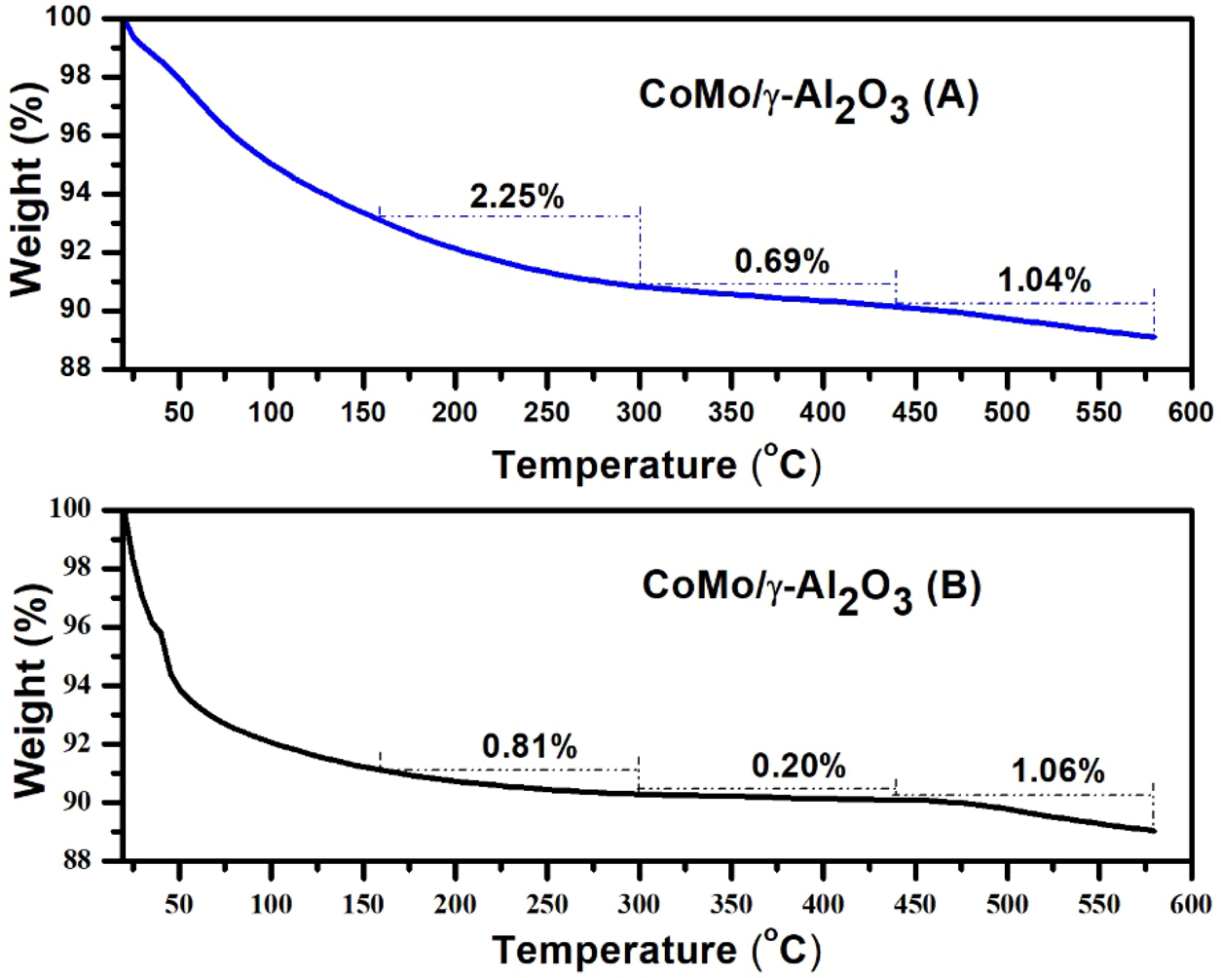
Temperature-programmed desorption curves of diethylamine for CoMo/γ-Al_2_O_3_ catalyst before (**A**) and after (**B**) sulphidation.

**Figure 4:**
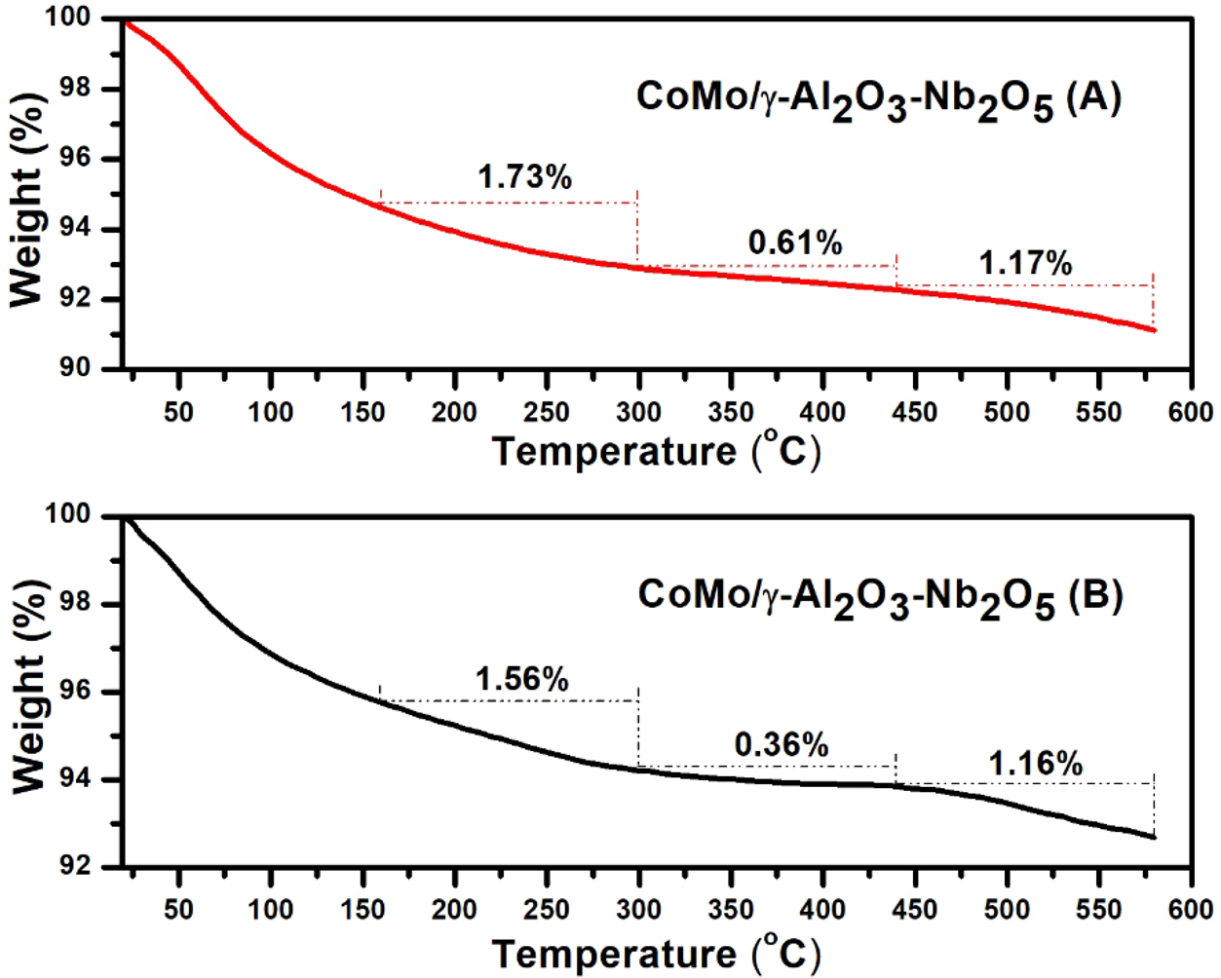
Temperature-programmed desorption curves of diethylamine for CoMo/γ-Al_2_O_3_ –Nb_2_O_5_ catalyst before (**A**) and after (**B**) sulphidation.

Temperature-programmed desorption curves of diethylamine are presented in Figs. 3 and 4 and the distribution of acidic sites of the catalysts, in terms of diethylamine weight loss, with increase in temperature from 20 to 600 °C are presented in Table 2.

**Table 2:** Acid strength distribution.

Catalyst	Weak acids centers meq/g		Medium acidic centers meq/g		Strong acidic centers meq/g		Total acidity meq/g	Total acidity meq/g
	A*	B*	A*	B*	A*	B*	A*	B*
CoMo/γ-Al_2_O_3_	0.308	0.111	0.094	0.027	0.142	0.145	0.544	0.283
CoMo/γ-Al_2_O_3_-Nb_2_O_5_	0.237	0.213	0.083	0.049	0.160	0.159	0.480	0.421

*A—samples before sulphidation.

*B—samples after sulphidation.

The total acidity of CoMo/γ-Al_2_O_3_ catalyst before sulphidation (0.544 meq DEA/g catalyst) was higher than that of CoMo/γ-Al_2_O_3_-Nb_2_O_5_ catalyst (0.480 meq/g) (Table 2). The γ-Alumina support has both Lewis and Brönsted acidity and is commonly used for HDS catalysts. It can be observed (Table 2) that after sulphidation, the acidity of CoMo/γ-Al_2_O_3_ catalyst decreased significantly compared to the acidity of the CoMo/γ-Al_2_O_3_-Nb_2_O_5_ catalyst. This behavior can be explain by the presence of niobium pentoxide acid which has both Lewis and Brönsted acidity (provided by Nb) due to the remaining –OH groups^11, 20^.

he distribution of acidic sites in terms of diethylamine weight loss with increase in.

temperature from 20 to 600 °C for the catalyst supports is presented in Fig. 1.

emperature-programmed desorption curves of diethylamine for CoMo/γ-Al.

### 2

#### O

### 3

catalyst before (A).

and after sulphurization (B.

Not only acidic strength, but also the acid types affect the HDS performances of the catalysts, since only appropriate amounts of Brönsted acid sites (B) and Lewis acid sites (L) can facilitate the adsorption of reactant molecules and the surface reactions. The amounts of Brönsted and Lewis acid sites of the catalysts, before and after sulphidation were calculated according to the Py-FTIR analyses.

**Figure 5:**
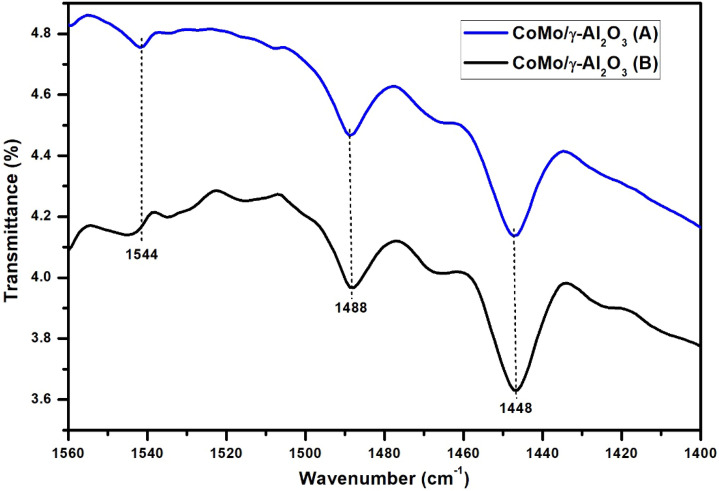
Pyridine IR absorption bands of CoMo/γ-Al_2_O_3_ catalyst before (**A**) and after (**B**) sulphidation.

**Figure 6:**
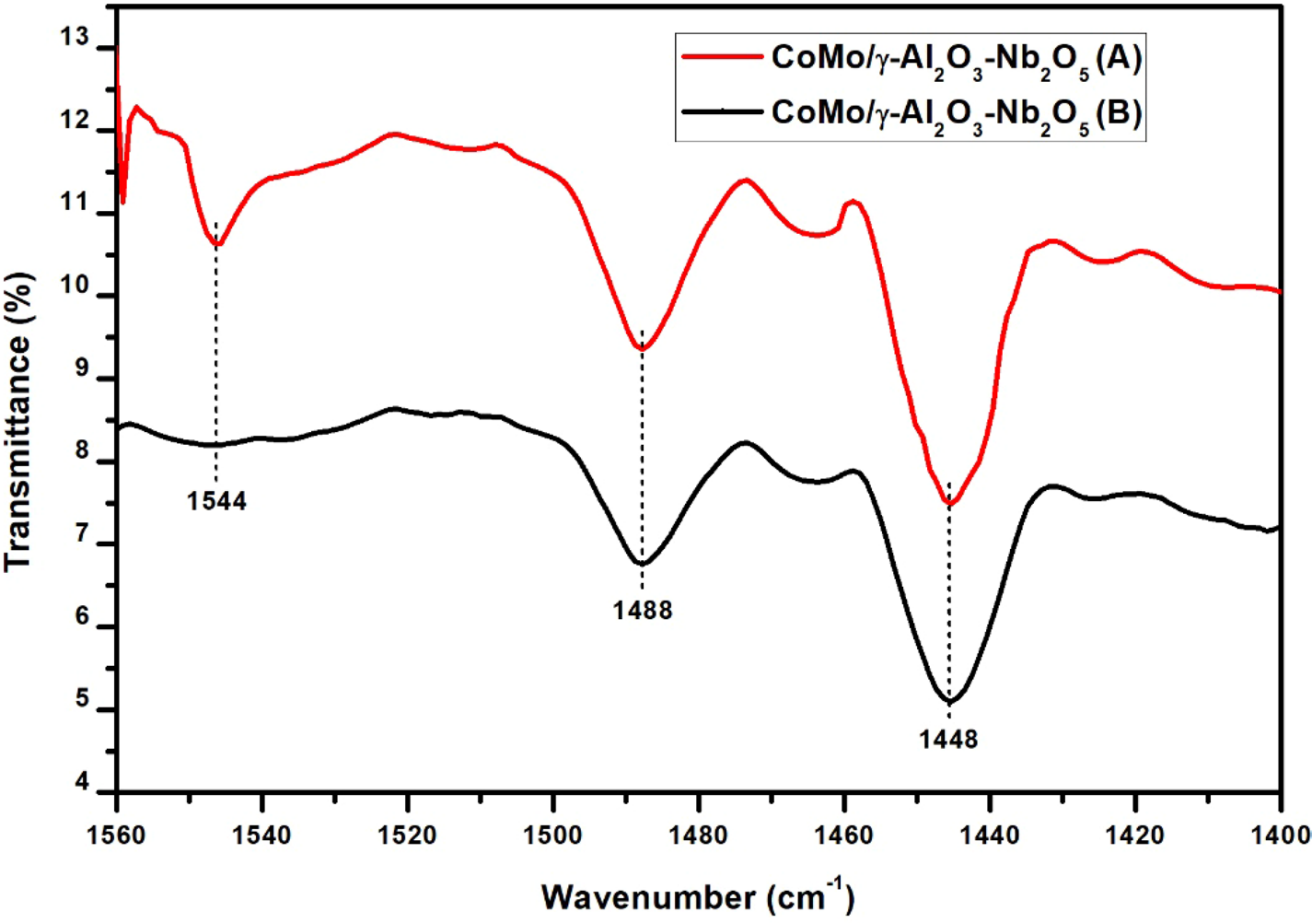
Pyridine IR absorption bands of CoMo/γ-Al_2_O_3_-Nb_2_O_5_ catalyst before (**A**) and after (**B**) sulphidation.

The pyridine-adsorbed FT-IR spectrum showed features in the region of 1400–1560 cm^−1^ due to the stretching vibrations of M–N (metal–nitrogen) and N–H (pyridinium ion). The IR vibration peak at 1544 cm^−1^ is ascribed to Brönsted acid sites and the IR vibration peak at 1448 cm^−1^ is assigned to Lewis acid sites (Figs. 5 and 6) and the band around wavenumber 1488 cm^−1^ is due to physically adsorbed pyridine^21, 22^. A significant amount of Lewis acid sites can be observed for CoMo/γ-Al_2_O_3_-Nb_2_O_5_ catalyst. It can be clearly seen that the use of Nb improve the Lewis acid sites of the CoMo/γ-Al_2_O_3_ catalyst. This may be because Nb atoms have a special outer electronic structure, which makes it exhibit more empty orbitals, leading to the formation of more Lewis acid sites.

After sulphidation the initial Lewis acidity increased and the Bronsted acidity of the catalysts decreased. The amount of Lewis acid sites, for CoMo/γ-Al_2_O_3_ catalyst after sulphidation, increased from 113.5 to 121.7 μmol·g^−1^ and for CoMo/γ-Al_2_O_3_-Nb_2_O_5_ catalyst, increased from 141.8 to 170.4 μmol·g^−1^. The amount of Bronsted acid sites decreased for both catalysts.

#### X-ray photoelectron spectroscopy (XPS)

**Figure 7:**
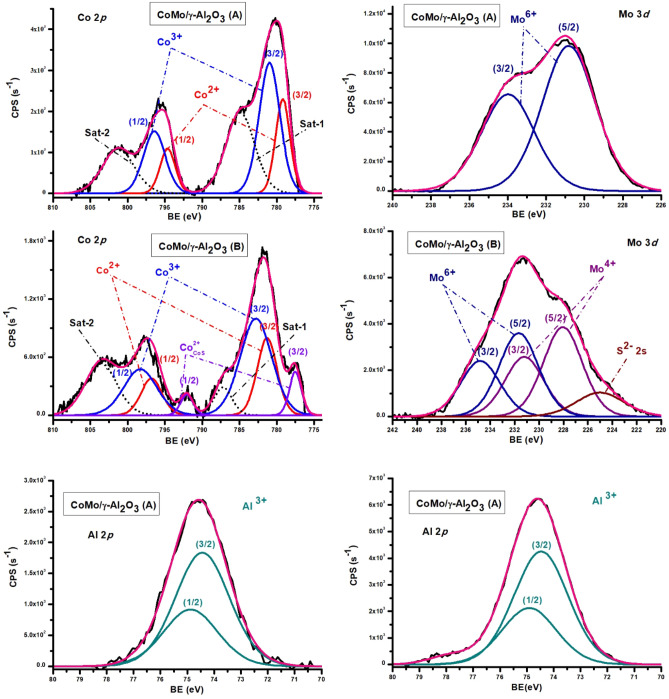
XPS Co 2p, Mo 3d, Al 2p and S 2 s spectra of CoMo/γ-Al_2_O catalyst before (**A**) and after (**B**) sulphidation.

The chemical species present on the catalysts surface were evaluated by XPS to explain the activity in HDS of thiophene, 2-ethylthiophene and benzothiophene. In CoMo/γ-Al_2_O_3_ catalyst, before and after sulphidation (Fig. 7), Co was present as Co^3+^ and Co^2+^ species. The binding energy of Co^3+^, with the Co 2p_3/2_ profile, was 780.97 eV (before sulphidation) and 782.79 eV (after sulphidation) which could be ascribed to Co_3_O_4_ and CoOOH^23^. Co^2+^ was present as CoO and CoAl_2_O_4_^24^ with Co 2p_3/2_ peak at 779.19 eV (before sulphidation) and 780.30 eV (after sulphidation). After sulphidation the Co 2p_3/2_ binding energy region showed a component at 777.52 eV which may be attributed to Co-S structures, but we are unable to give the exact composition for such a species^25^. The deconvolution of Co 2p spectra also included the two characteristic satellite features. The Mo 3d spectra of catalysts before and after sulphidation, indicated Mo^6+^ species, with the Mo 3d_5/2_ peak at 230.81 eV (before sulphidation) and 231.66 eV (after sulphidation), which could be attributed to MoO_3_^26, 27^. After sulphidation MoS_2_ was formed^28^, shown by the presence of the Mo 3d_5/2_ peak at 228.06 eV. As expected after sulphidation, in the Mo 3d spectra the S 2 s peaks appeared, corresponding to sulfide and sulfate components. From the calcination step of the catalyst preparation Co and Mo aluminates or double oxides containing Co and Mo could also be formed^29–31^.

The S 2p_3/2_ binding energy region showed a component at 160.69 eV and S 2 s at 225.08 eV that was characteristic of sulfide (S ^2−^) species. Aluminium in γ-Al_2_O_3_ , before and after sulphidation had a peak at 74.44 eV (Al 2p_3/2_), and the oxygen corresponding to metal oxides had the O 1 s peak at 530 eV and 531.4 eV.

**Figure 8:**
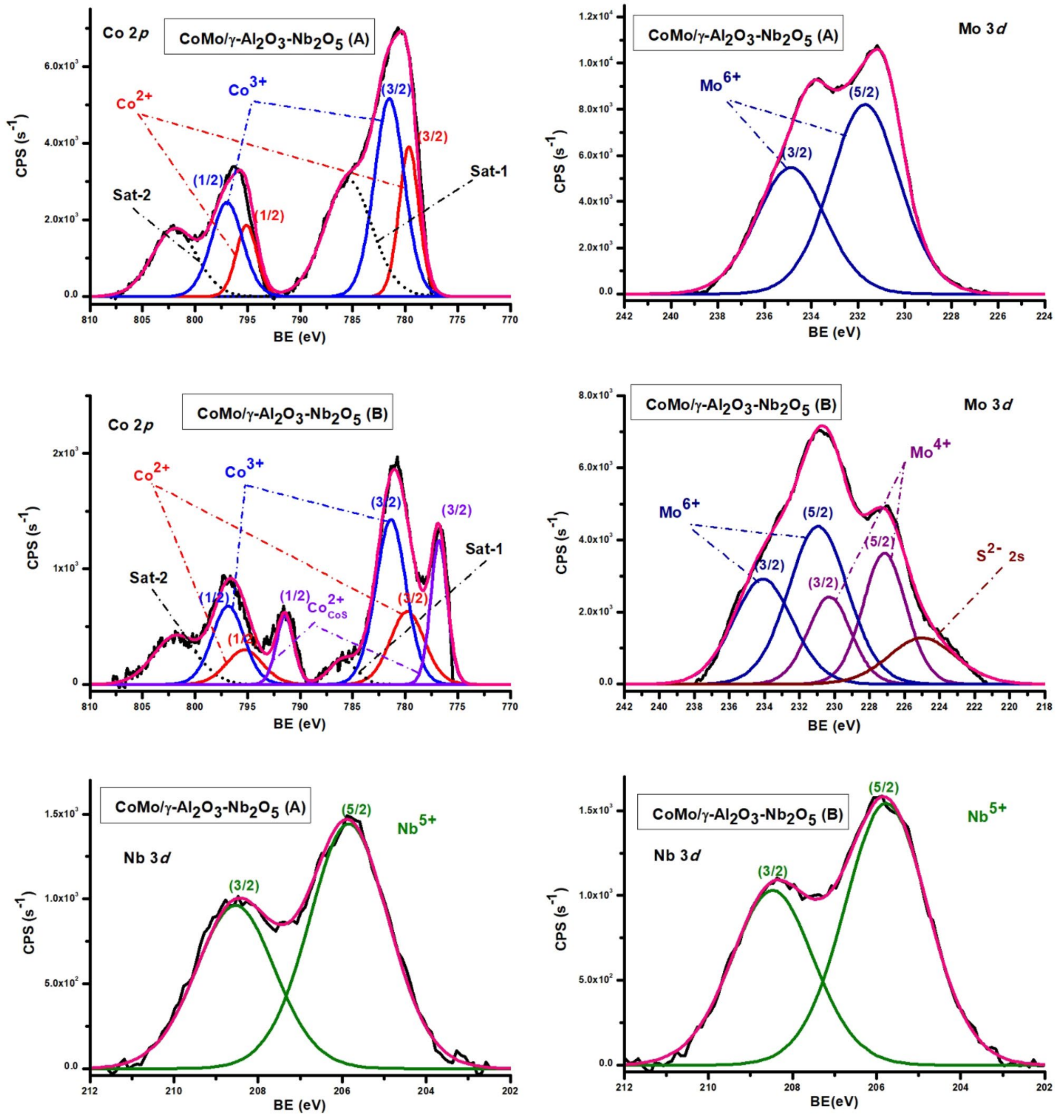
The XPS spectra of CoMo/γ-Al_2_O_3_-Nb_2_O_5_ catalyst before and after sulphidation.

The XPS spectra of CoMo/γ-Al_2_O_3_-Nb_2_O_5_ catalyst (Fig. 8) indicated the presence of Mo^6+^ with characteristic Mo 3d_5/2_ peak at 231.7 eV before sulphidation and 230.94 eV after sulphidation. MoS_2_ species, after sulphidation, were confirmed by Mo 3d_5/2_ peak at 227.15 eV and by S 2 s and S 2p_3/2_ peaks at 224.96 eV and 160.33 eV. Similar to the CoMo/γ-Al_2_O_3_ catalyst, Co had specific peaks corresponding to Co^3+^ and Co^2+^ species and after sulphidation also the Co 2p_3/2_ peak at 776.79 eV, corresponding to metallic cobalt. The peak at 205.86 eV was characteristic to Nb 3d_5/2_ associated to Nb_2_O_5._ After sulphidation the binding energy was the same, indicating that niobium remains as Nb_2_O_5_.

According to our results, Co^2+^ and Mo^4+^ species are present in the sulfide form on both catalysts surfaces. In addition, there are also found oxidic species arising from incomplete reduction and sulphidation. The ratios between doublet components line widths were set between 1 and 1.1.^32^.

The theoretical mass percentage of Co, Mo and Nb reported to the total metal species (Co + Mo or Co + Mo + Nb), are as follow: for CoMo/γ-Al_2_O_3_ catalyst: Co (33.33%) and Mo (66.66%) and for CoMo/γ-Al_2_O_3_-Nb_2_O_5_ catalyst: Co (25.8%), Mo (51.61%), Ni (5.405%), and Nb (22.5%). The mass percentage calculated from the curve fitting analysis is presented in Table 3. M% represents mass percentage of different metal species (Me_i_), reported to the total metal species, resulting from the curve fitting analysis.

**Table 3:** Binding energies (eV) and mass percentage from fitting curve analysis, for metal species of catalysts before (A) and after sulphidation (B).

Catalyst		Co^3+^ 2p_3/2_ (Co_2_O_3_, CoOOH)	Co^2+^ 2p_3/2_ (CoO)	Co^2+^ 2p_3/2_ (Co_x_S_y_)	Mo^6+^ 3d_5/2_ (MoO_3_)	Mo^4+^ 3d_5/2_ (MoS_2_)	Nb^5+^ 2p_3/2_ (Nb_2_O_5_)
CoMo/γ-Al_2_O_3_ (A)	BE (eV)	780.97	779.19	–	230.81	–	–
	M%	18.213	12.371	–	69.415	–	–
CoMo/γ-Al_2_O_3_ (B)	BE (eV)	782.79	780.30	777.52	231.66	228.06	–
	M%	11.317	7.579	11.145	21.262	48.696	–
CoMo/γ-Al_2_O_3_-Nb_2_O_5_ (A)	BE (eV)	781.51	779.66	–	231.70	–	205.84
	M%	18.306	8.196	–	54.644	–	18.852
CoMo/γ-Al_2_O_3_-Nb_2_O_5_ (B)	BE (eV)	781.35	779.80	776.79	230.94	227.15	205.68
	M%	7.202	3.324	14.958	1.108	55.401	18.105

The presence of niobium oxide in the catalytic support had a positive effect in the sulphidation degree. The sulphidation degree for each metal species was calculated from the XPS analysis, resulting in Co (37.1%), and Mo (69.6%) in the CoMo/γ-Al_2_O_3_ catalyst and Co (58.7%), and Mo (98%) in the CoMo/γ-Al_2_O_3_-Nb_2_O_5_ catalyst.

Peak assignment was carried out according to literature sources^26, 27, 33^. All the values of the binding energies listed here are generally subject to ± 0.3 eV variations as a result of C 1 s positioning errors.

### Hydrodesulfurization of thiophene, 2-ethylthiophene and benzothiophene as single component and their ternary mixture

#### CoMo/γ-Al_2_O_3_ catalyst

**Figure 9:**
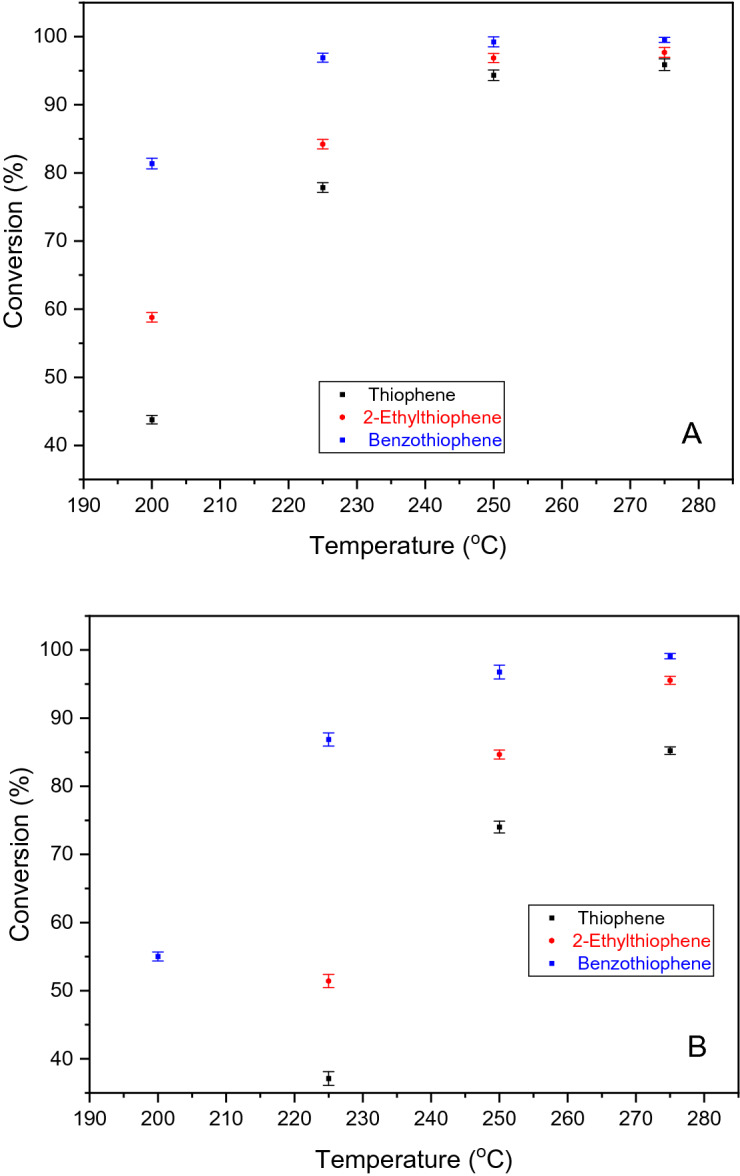
Conversion with temperature over CoMo/γ-Al_2_O_3_ catalyst. (**A**) Single sulfur compound HDS (T, 2-ET, BT); (**B**) mixture of sulfur compounds HDS (T + 2-ET + BT).

Conversions increased continuously over the investigated temperature range (200 – 275 °C), both for HDS of individual components (T, 2-ET and BT) (Fig. 9A) and their ternary mixture (Fig. 9B); the influence of temperature was more pronounced up to 250 °C. A mutual inhibiting effect between the aromatic sulfur compounds was observed in HDS of ternary mixture, as conversions were lower for the mixed HDS compared to the HDS single component system. The individual compound HDS led (at 200 °C) to a thiophene conversion of 44.27%, whereas in the ternary mixture was 9.92%. Same effect was observed at 275 °C, where the individual HDS of thiophene provided a conversion of 95.98%, whereas in the mixture was 85.52%.

For 2-ethylthiophene, the conversion was 58.81% at 200 °C, 96.92% at 250 °C, and reached 97.85% at 275 °C (Fig. 9A). In the ternary mixture (Fig. 9B), the conversion of 2-ethylthiophene increased from 17.20% at 200 °C, to 84.98%, at 250 °C, and reached 95.75% at 275 °C.

The conversion of benzothiophene (individual HDS), increased from 81.63 to 97.02% in the temperature range of 200–225 °C, compared to that of 225–275 °C, when the conversion increased less, from 97.02 to 99.77%. In the mixture, the conversion of benzothiophene at 200 °C was 55.17%, 86.90% at 225 °C, and 99.11% at 275 °C.

**Figure 10:**
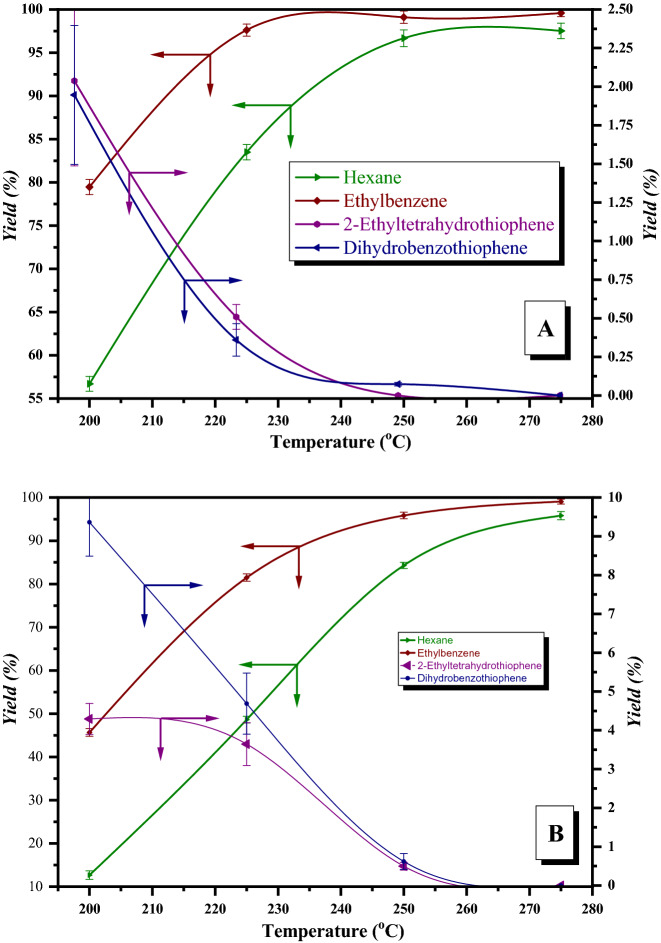
Product yields with temperature over CoMo/γ-Al_2_O_3_; (**A**) single sulfur compound HDS (T, 2-ET, BT); (**B**) mixture of sulfur compounds HDS (T + 2-ET + BT).

The HDS of aromatic sulfur compounds proceeds through successive and parallel reactions of hydrogenolysis of C–S bonds. The resulting final products did not contain sulfur and H_2_S, through partial or total hydrogenation of these aromatic sulfur compounds^34–36^, with the formation of other heterocyclic sulfur compounds, unsaturated or saturated. Figure 10 shows the yield variations of the liquid reaction products, with temperatures, over the CoMo/γ-Al_2_O_3_ catalyst. For thiophene HDS, the concentrations of the intermediate products (dihydrothiophene and tetrahydrothiophene), were less than 0.2% and therefore the variations of the yields of these products were not graphically represented, which means that for thiophene HDS over tested CoMo catalysts, the hydrogenation reactions (with the formation of saturated or unsaturated cyclic sulfur compounds) are less than of the benzothiophene HDS process.

High temperatures favored the formation of the final hydrogenated products (Fig. 10). For example, in the case of 2-ethylthiophene HDS, at 200 °C, the yield of 2-ethyltetrahydrothiophene (intermediate product) was 2.01% and the yield of hexane (final product) was 56.80%. By increasing the temperature, final product conversion rates were higher. At 275 °C, the yield of hexane reached 97.85% (Fig. 10A). The same behavior was observed in HDS of the equimolar mixture for aromatic sulfur compounds (Fig. 10B).

The HDS of benzothiophene, produces dihydrobenzothiophene as an intermediate product and ethylbenzene as the final product. The yield in dihydrobenzothiophene decreased from 1.94% at 200 °C to 0% at 275 °C, and ethylbenzene yield increased from 79.80% to 99.78%. In the HDS equimolar mixture of T, 2-ET and BT, (Fig. 10B), the product yields of BT hydrodesulphurization had lower values than the yields of these products, which resulted in the HDS of pure benzothiophene. The tendency to convert intermediate products into final products was maintained.

The results presented in Fig. 10 are supported by the work of^37^ reporting that sulfur removal from thiophene is slower than from benzothiophene. Same reactivity order is reported by Kilanowski et al.^38^.

#### CoMo/γ-Al_2_O_3_-Nb_2_O_5_ catalyst

**Figure 11:**
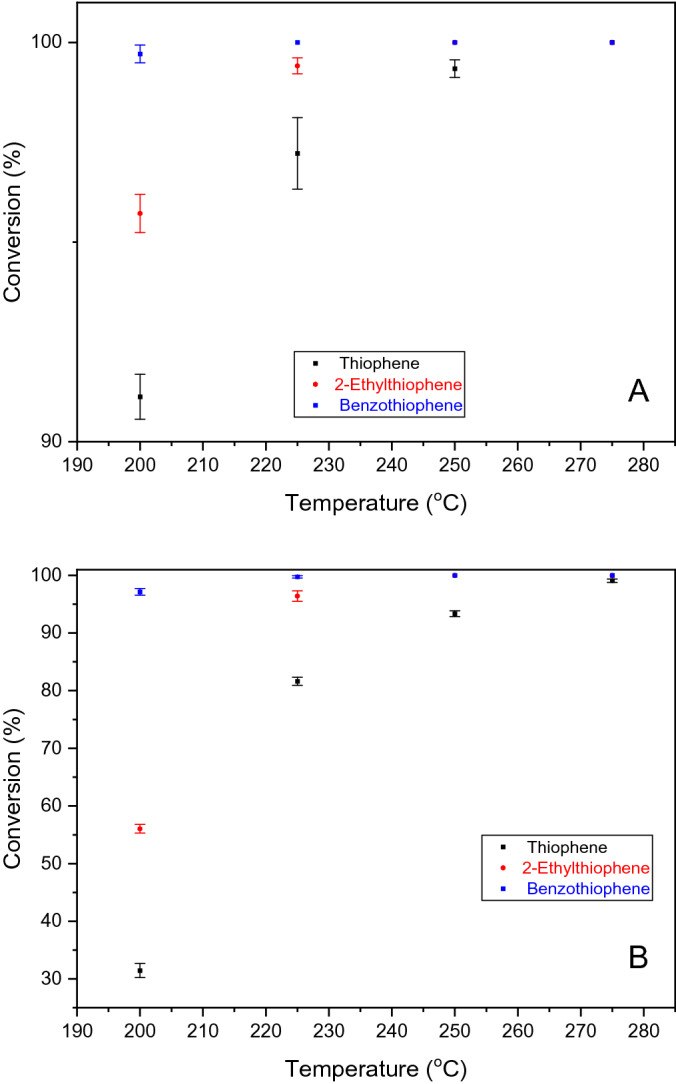
Conversion with temperature over CoMo/γ-Al_2_O_3_-Nb_2_O_5_; (**A**) single sulfur compound HDS (T, 2-ET, BT); (**B**) mixture of sulfur compounds HDS (T + 2-ET + BT).

CoMo/γ-Al_2_O_3_-Nb_2_O_5_ catalyst had a higher activity in hydrodesulphurization process of T, 2-ET and BT, their conversions being higher than 90% for single component HD in the studied temperature range. At 200 °C, the conversion of T was already 91.27% and reached 100% at 275 °C. The conversion of 2-ET was 96.15% at 200 °C and 100% at 275 °C, and the conversion of BT had reached 100% at temperatures higher than 200 °C (Fig. 11A).

For the mixed species, the conversion of sulfur compounds was smaller compared to the conversion of the single component hydro-sulfurization. At 200 °C, the conversions were 31.73% (T), 56.33% (2-ET) and 97.37% (BT). However, at higher temperatures (275 °C), the conversion for each component was closer to the conversion obtained in the single component hydro-sulfurization (Fig. 11B).

**Figure 12:**
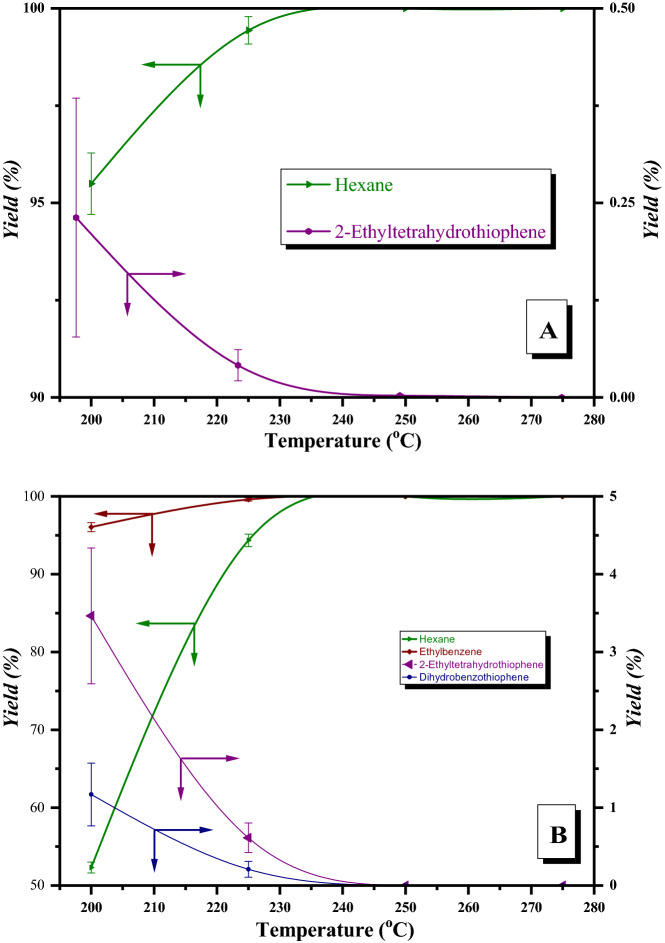
Product yields with temperature over CoMo/γ-Al_2_O_3_–Nb_2_O_5_; (**A**) single thiophenic compound HDS (T, 2-ET, BT); (**B**) mixture of thiophenic compounds HDS (T + 2-ET + BT).

The influence of temperature on the yields of reaction products for a single component and mixture of T, 2-ET and BT hydrodesulphurization, over CoMo/γ-Al_2_O_3_-Nb_2_O_5_ catalyst is presented in Fig. 12.

The CoMo/γ-Al_2_O_3_-Nb_2_O_5_ catalyst favored the formation of the sulfur-free products. For benzothiophene HDS, dihydrobenzothiophene was not identified and for 2-ethylthiothiothiophene HDS, the yield of 2-ethyltetrahydrothiophene was below 1%.

For the HDS mixture of aromatic sulfur compounds (Fig. 12B), the yield of intermediate products increased slightly compared to the yields obtained in single component HDS. For example, the yield of 2-ethyltetrahydrothiophene was 3.70% at 200 °C and decreased with increasing temperature (Fig. 12B). However, the yield of hexane increased, from 52.64% at 200 °C to 100% at 275 °C. The yield of dihydrobenzothiophene was 1.11% at 200 °C and decreased with increasing temperature to 0%.

The sulfur concentration (ppm) after the HDS process is listed in Table 4. At 275 °C, the sulfur is completely removed from all sulfur compounds in single component HDS and is reduced to 7.23 ppm in a mixture of sulfur compounds HDS.

**Table 4:** Sulfur concentration (ppm) after HDS of thiophene, 2-ethylthiophene and benzothiophene, at 275 °C.

Catalyst/Sulfur compounds	CoMo/γ-Al_2_O_3_	CoMo/γ-Al_2_O_3_-Nb_2_O_5_
	Sulfur, ppm	
Thiophene (T)	95.78	0
2-Ethyl-thiophene (ET)	51.34	0
Benzothiophene (BT)	5.45	0
Mixture of T-ET-BT	155.74	7.23

From the experimental results, one can note that:

1. HDS reactivity of the tested sulfur compounds decreased in the following order: BT > 2-ET > T;
2. CoMo/γ-Al_2_O_3_-Nb_2_O_5_ catalyst was more active in HDS for the tested sulfur compounds compared to that of the CoMo/γ-Al_2_O_3_ catalyst;
3. There was a strong inhibition effect in the HDS of a ternary mixture of T, 2-ET and BT compared to that of a single component HDS species. The inhibition effect was more pronounced for T, than for 2-ET and BT. The inhibition effect decreased with increasing temperature and was less pronounced over the CoMo/γ-Al_2_O_3_-Nb_2_O_5_ catalyst. This behavior can be explained by two factors: electronic and steric components. The molecular size of the sulfur compounds (molecular critical diameter) decreases in the order benzothiophene > 2-ethylthiophene > thiophene. The size is between 4.65 Å for thiophene^39^ and 6 Å for benzothiophene^40^. Consequently, the adsorption of thiophene on active sites of the catalyst should be more pronounced than that of benzothiophene, if the smaller volume of the thiophene molecule is considered.

The thiophene compounds are classified as aromatic because of their high resonance energy (29 kcal/mol for thiophene) and their properties, but the aromatic character decreases in the case of condensed rings, as in the case of benzothiophene^41^. Sulfur participates with two electrons in the formation of the aromatic electron sextet and the thiophene nucleus of the aromatic sulfur compounds shows basicity due to the six π electrons delocalized on the five-membered heterocyclic compound^41^. The molecular adsorption on the acidic sites of the catalyst is favored if the basicity of the aromatic nucleus. In the series of investigated compounds, the basicity increases in the order of thiophene < 2-ethylthiophene < benzothiophene. The higher basicity of benzothiophene is due to the weaker delocalization of π electrons on the two condensed aromatic nuclei. Subsequently, these electrons interact more strongly with the acidic centers of the catalyst, compared to that of thiophene, which has a single aromatic nucleus and a stronger aromatic character. 2-Ethylthiophene has higher basicity in the nucleus than thiophene due to the ethyl group which stabilizes the positive charge that appears in the nucleus, as a result of the interactions of the aromatic ring—acid centers in the catalyst. The higher reactivity of benzothiophene proves that the HDS process at the investigated parameters is influenced more by the electronic factors that favor the adsorption of sulfur compounds on the active centers of the catalyst than by the steric factors. The influence is also explained by the fact that the pore volume of the catalyst, 3.27 nm, is larger than the size of the molecules of the studied sulfur compounds^40^. High HDS reactivity of benzothiophene, despite the larger molecular volume, has also been reported in other the studies^40^. The higher the rate of the hydrogenation reaction of benzothiophene compared to thiophene, results in the higher reactivity of benzothiophene compared to that of 2-methylthiophene and 3-methylthiophene (Brunet et al.^18^). The relative reactivity of sulfur aromatic compounds depends on their structure.

All three aromatic sulfur compounds react more slowly in HDS where the mixture is more influenced by thiophene than by 2-ethylthiophene and benzothiophene. This inhibition effect may be explained by the competitive adsorption between the three aromatic sulfur compounds on the active sites of the catalyst. The adsorption of each compound is delayed by the presence of the other two. Since the reactivity of the sulfur compounds is different, the inhibition effect is the lowest in the case of the sulfur compound with the highest in the HDS reaction with benzothiophene.

At higher temperatures (approx. 275 °C), the inhibition effect decreases considerably or is no longer present because the HDS reaction rate for all sulfur compounds increases more compared to the reaction rate at lower temperature (ex. 200 °C). Similarly, it was observed that the inhibition effect is attenuated on CoMo/γ-Al_2_O_3_-Nb_2_O_5,_ due to better textural characteristics and higher acidity, compared to the CoMo/γ-Al_2_O_3_ catalyst.

It should be pointed out that under the conditions of industrial HDS, the petroleum fractions contain a mixture of sulfur compounds, depending on the distillation interval of these fractions; thus, it may be an important deviation from the additivity to HDS of mixtures of sulfur compounds in comparison with the individual HDS of these compounds studied in the laboratory.

## Conclusions

The results presented in this paper showed that the CoMo/γ-Al_2_O_3_-Nb_2_O_5_ catalyst is more active in the HDS reaction of aromatic sulfur compounds than CoMo/γ-Al_2_O_3_ catalyst due to the higher specific surface area and higher acidity of the niobium catalyst after sulphidation.

The reactivity in the HDS reaction decreases in the order of benzothiophene > 2-ethylthiophene > thiophene, because the adsorption of the molecules on the acid centers of the catalyst is favored if the basicity of the aromatic nucleus is higher. Higher HDS reactivity of benzothiophene under the experimental conditions, proves that the process is influenced by the electronic factors that favor the adsorption of sulfur compounds on the active sites of the catalyst and not by the steric factors.

The experimental data has shown that for HDS of an equimolar mixture of aromatic sulfur compounds, under the experimental conditions (200–275 °C, 30 bar and LHSV 2 h^−1^), there is a strong mutual inhibition effect compared to that of the single component HDS species. The effect is more pronounced at lower temperatures (200–250 °C), thiophene being more influential, than 2-ethylthiophene and benzothiophene. The inhibition effect decreases with increasing catalyst activity.

The inhibition effect can be explained by the competitive adsorption between the three aromatic sulfur compounds on the active sites of the catalyst.
